# Brief reviews

**DOI:** 10.1590/S1980-57642011DN05010012

**Published:** 2011

**Authors:** Sonia Maria Dozzi Brucki

**Affiliations:** 1 Behavioral and Cognitive Neurology Unit, Department of Neurology, and Cognitive Disorders Reference Center (CEREDIC). Hospital das Clínicas of the University of São Paulo School of Medicine, São Paulo SP, Brazil.

## INCIDENCE AND SUBTYPES OF EARLY-ONSET DEMENTIA IN A GEOGRAPHICALLY DEFINED GENERAL POPULATION

**Garre-Olmo et al. Neurology 2010;75:1249-1255.**

Early-onset dementia (EOD) is based on a cutoff for dementia with an age at onset of younger than 65 years. Epidemiological studies on the prevalence of EOD are relatively scarce yet welcome given the disease’s economic, social, and clinical impact for patients and caregivers. This study, carried out in Girona (Spain), evaluated a clinical registry of new dementia cases in seven hospitals between 2007 and 2009.

A total of 6.9% of the 2083 patients registered in this three-year period were diagnosed as EOD; with an incidence of 6 cases/100,000 person-years for the 30-49 years age group. The most frequent cause was Alzheimer’s disease (42.4%), followed by secondary dementias (18.1%), vascular dementia (13.8%) and frontotemporal dementia (9.7%). There was no gender predominance, and greater frequencies of EOD patients were married, living in their own house, and were more cognitively impaired. This report provided important information about EOD, which differed to other previously published data from hospital-based studies.

## REGIONAL BRAIN ATROPHY AND FUNCTIONAL DISCONNECTION ACROSS ALZHEIMER’S DISEASE EVOLUTION

**Gili et al. Journal of Neurology Neurosurgery and Psychiatry 2011;82:58-66.**

Clinical manifestations of AD are probably due to regional gray matter loss and to abnormal functional integration of brain regions. Default mode network (DMN) comprises posterior cingulate cortex (PCC), inferior parietal cortex and medial prefrontal cortex (mPFC), where these areas exhibit spontaneous brain activity during rest, and can be modulated by cognitive tasks. These regions exhibit a coherent pattern of activation (functional connectivity). The authors investigated gray matter (GM) atrophy and DMN disconnection as well as their correlation with cognitive decline, in 10 healthy elderly subjects (HS), 10 amnestic mild cognitive impairment (a-MCI) patients and 11 AD patients.

AD and a-MCI patients showed a pattern of brain disconnection between the PCC and mPFC and the rest of the brain, seen on functional MRI. There was a regional GM reduction (by voxel based morphometry) which was more spread in AD patients than a-MCI patients and controls, with more specific atrophy to hippocampus and mPFC bilaterally, compared to a-MCI and controls. PCC showed reduced connectivity in a-MCI patients without GM atrophy, results which suggests that disconnection precedes GM atrophy in the PCC, and that atrophy of this latter structure could be a marker for conversion from MCI to AD.

## CLINICAL EXPERIENCE AND LABORATORY INVESTIGATION IN PATIENTS WITH ANTI-NMDAR ENCEPHALITIS

**Dalmau et al. Lancet Neurology 2011;10:63-74.**

A complete review of a clinical experience with 400 anti-NMDAR encephalitis patients is presented by the authors. This syndrome is characterized by antibodies against the NR1 subunit of the NMDA receptor. The authors described a flourishing clinical picture, and reported that this syndrome is probably underdiagnosed. Some points deserve highlighting: about 70% of patients have prodromic symptoms such as headache, fever, nausea, vomiting, diarrhea, or upper respiratory-tract symptoms. Typically, within less than two weeks, patients develop psychiatric, cognitive symptoms, and disintegration of language. Motor and complex seizures develop at early stages. It is important to remember that this syndrome can occur in children, and may be associated with ovarian teratoma, and testicular tumor. Many other aspects are discussed including associated tumors and triggers of the immune response, making this report a reference on this issue. The publication also incorporates a discussion about treatment with immunosuppressive drugs and outcome.

## THE ROLE OF CLUSTERIN, COMPLEMENT RECEPTOR 1, AND PHOSPHATIDYLINOSITOL BINDING CLATHRIN ASSEMBLY PROTEIN IN ALZHEIMER’S DISEASE RISK AND CSF BIOMARKERS LEVELS.

**Brit-Maren et al. Archives of General Psychiatry 2011; 68:207-213.**

This study was performed in 1245 families, with 2654 AD patients and 1175 unaffected relatives in the USA, and one center in Germany that carried out a genetic study in 214 AD patients and 211 controls. There are five genetic variants in CLU, CR1, and PICALM, but 3 investigated loci are associated with risk for AD. Of all the markers, only RS541458 in the PICALM gene was shown to have an effect on CSF protein levels. The AD risk allele is associated with decreased CSF AB 42 levels.

## USE OF FLORBETAPIR-PET FOR IMAGING β- AMYLOID PATHOLOGY

**Clark et al. JAMA 2011;305:275-283.**

Biomarkers can be used to enable early identification of AD patients or those who are at risk for developing AD. A previous biomarker, PET with PIB (Pittsburg compound B), was the first ligand to visualize β-amyloid in living patients. But its short half-life, about 20 minutes, limits its use. Florbetapir is a new ligand to the amyloid, with a greater half-life and rapid entry to the brain. The study compares visual images obtained by PET of patients and controls against those of 29 autopsied individuals. Visual interpretation of the flobetapir-PET images and mean quantitative estimates of cortical uptake were correlated with presence and quantity of β-Amyloid pathology at autopsy. This method obtained a sensitivity of 93% and a specificity of 100%, suggesting an accurate and reliable assessment of amyloid pathology in life.

## BRAIN BIOPSY IN DEMENTIA: CLINICAL INDICATIONS AND DIAGNOSTIC APPROACH

**Schott et al. Acta Neuropathol 2010;120(3):327-341.**

This paper reviews the use of brain biopsy as a diagnostic tool in dementia, considering the most recent developments in biomarkers and neuropathological diagnosis, as well as the better understanding of cognitive impairment in autoimmune/corticosteroid-responsive disorders. Following the paper published in 2005 (Warren et al., 2005), the authors discuss the changes in diagnostic yield and relevance to treatment of brain biopsy in rapidly progressive or atypical dementia. Comparing the previous (1989-2003, with 90 biopsies) and current (2004-2009, with 19 cases) series reveals that, whilst there was a rise in frequency at which a specific diagnosis was reached (from 57% to 74%), the biopsy results modified therapy in 11% of cases for both series. In the former series, Alzheimer’s disease was the most frequent diagnosis but was found in none of the biopsies performed from 2004-2009 (when Creutzfeldt-Jakob disease became the most frequent diagnosis). Complications occurred in 11-21% of procedures, and no deaths were reported. The authors conclude that although the use of new biomarkers, neuroimaging and antibodies dosage in clinical practice may reduce the need for invasive diagnostic procedures, cerebral biopsy is valuable in some cases as it may provide treatment-changing information. An algorithm for the clinical rationale justifying patient candidates for cerebral biopsy is provided.

